# Supplementary material for: Gravimetry through non-linear optomechanics
Source: Nat Commun. 2018 Sep 11;9:3690. doi: 10.1038/s41467-018-06037-z (PMC6133990; doi:10.1038/s41467-018-06037-z)
Supplement: Supplementary file 1 — Supplementary Information [file 41467_2018_6037_MOESM1_ESM.pdf]

Supplementary Information

Sofia Qvarfort,* Alessio Serafini, P. F. Barker, and Sougato Bose
Department of Physics and Astronomy, University College London, Gower Street, WC1E 6BT London, United Kingdom
(Dated: August 23, 2018)

Supplementary Note 1

In this section, we will show that the Fisher information remains unchanged at $t = 2\pi$ if the initial state is a thermal coherent state. Experimentally, this corresponds to a system that has not been cooled to the ground state.

A general thermal mixed state is given by

$$\rho_{\mathrm{Th}}(\xi, \beta) = \frac{1}{Z}\sum_{m=0}^{\infty} e^{-\hbar\beta\omega} D(\xi)\,|m\rangle\,\langle m|\,D^\dagger(\xi), \qquad (1)$$

where $D(\xi) = e^{\xi b^\dagger - \xi^* b}$ is a Weyl displacement operator and β is now the inverse temperature rather than the coherent state parameter. The partition function is given by $Z = \frac{1}{1-e^{-\hbar\beta\omega}}$. We must now show that the initial state $\rho(t=0) = |\alpha\rangle\langle\alpha| \otimes \rho_{\mathrm{Th}}$ still decouples under the dynamics. Applying the time-evolution operator in Eq. (6) gives the state

$$\begin{aligned}\rho(t) = &\sum_{n,n'} \frac{\alpha^n (\alpha^*)^{n'}}{\sqrt{n!n'!}} e^{i[\bar{k}^2(n^2-n'^2)-2\bar{k}\bar{g}(n-n')]\tau}\,|n\rangle\,\langle n'| \\ &\times \frac{1}{Z}\sum_m e^{-\hbar\beta\omega} e^{[(\bar{k}(n-n')-\bar{g})\eta\xi^* e^{it} - (\bar{k}(n-n')-\bar{g})\eta^*\xi e^{-it}]/2} \\ &\times e^{(\varphi_n(t)b^\dagger - \varphi_n^*(t)b}\,|m\rangle\,\langle m|\,e^{\varphi_n^*(t)b - \varphi_n(t)b^\dagger}, \qquad (2)\end{aligned}$$

with $\varphi_n(t) = (\bar{k}n - \bar{g})\eta + \xi e^{-it}$. Now, at $t = 2\pi$, we know that $(\bar{k}n - \bar{g})\eta + \xi e^{-it} = \xi$, and $\eta = 1 - e^{-it} = 0$, which means that the above state simplifies to

$$\begin{aligned}\rho(t) = &\sum_{n,n'} \frac{\alpha^n (\alpha^*)^{n'}}{\sqrt{n!n'!}} e^{i[\bar{k}^2(n^2-n'^2)-2\bar{k}\bar{g}(n-n')]2\pi}\,|n\rangle\,\langle n'| \\ &\otimes \frac{1}{Z}\sum_m e^{-\hbar\beta\omega} e^{\xi b^\dagger - \xi^* b}\,|m\rangle\,\langle m|\,e^{\xi^* b - \xi b^\dagger}, \qquad (3)\end{aligned}$$

where we can see that the oscillator has returned to its original state and has become completely decoupled. The cavity state has the same form as in Eq. (7) and so the resulting Fisher information will be the same as the one we calculated for coherent states in Eq. (11).

Supplementary Note 2

In the main text we computed the classical Fisher information (CFI) for pure states using the expression in Eq. (14).

Let us now investigate the effects of the parameters $\bar{k}$ and $\bar{g}$ on the CFI. In the main text, we plotted the values of I_{F} at $t = 2\pi$. For small enough $\bar{k}$, the Fisher information is not entirely suppressed for values $t \neq 2\pi$. If for $\bar{k} = \bar{g} = \alpha = 1$ we measure the position quadrature (with $\lambda = 0$) instead of momentum, we find that the Fisher information peaks just before $t = 2\pi$. This can be seen in Supplementary Figure 1a. However, comparing this value with the QFI at the same time ($t = 5.82$) shows that it is much lower at $I_{\mathrm{F}} = 2.37$ ms^{-2} versus $QFI = 628$ ms^{-2}. The numerical value in the plot is $\bar{I}_{\mathrm{F}} = 228$, but recall that we have to add the dimensionful prefactor of $\cos^2\theta m/(2\hbar\omega_m^2)$, which for $\bar{g} = 1$ becomes just $1/g^2$. Thus we see clearly that the homodyne measurement is optimal only at $t = 2\pi$.

We showed in Eq. (17) that the CFI is independent of $\bar{g}$ and scales with $\bar{k}^2$ at $t = 2\pi$. To emphasize this point, we here provide some additional computations of the CFI for different values of $\bar{k}$ and $\bar{g}$. Note that we are again computing the dimensionless part of I_{F} in Eq. (14), leaving out the prefactor $\cos^2\theta m/(2\hbar\omega_m^3)$ for clarity.

Supplementary Figure 2b shows the CFI for $\bar{k} = 1, 2, 3$ with $\bar{g} = 1$, and Supplementary Figure 2d shows its behaviour for $\bar{g} = 1, 2, 3$ with $\bar{k} = 1$. As expected, the CFI scales with $\bar{k}^2$ at $t = 2\pi$. We also note some additional oscillations near the peak as $\bar{k}$ becomes larger. For larger values of $\bar{g}$, we see only marginal changes in the behaviour of the function in the region around $t = 2\pi$.

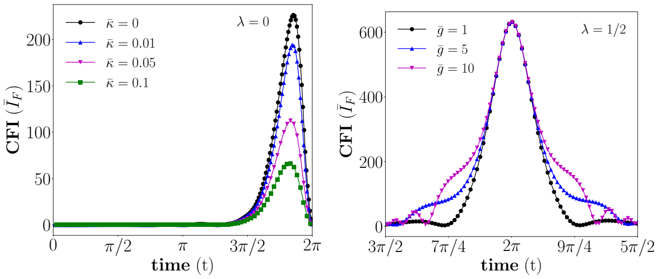

(a) (b)

Supplementary Figure 1: Plots showing the Fisher information for a homodyne measurement with (a) $\lambda = 0$, $\bar{k} = \bar{g} = 1$ with various decoherence rates, and (b) the Fisher information for $\lambda = \pi/2$ with $\bar{k} = 1$ and $\bar{g} = 1, 5, 10$.

* sofia.qvarfort.15@ucl.ac.uk

Supplementary Note 3

In the main text we presented a simplified noise model to estimate the Fisher information obtained from performing measurements on the leaking photons. The model is limited in its application because it involves a fully unitary process between the system and the environment. In other words, at some later time t, all information about g will be transferred back from the vacuum state into the coherent cavity state, a clearly unphysical process. Therefore, we limit ourselves to small values of γ, which also ensures the stability of the simulations.

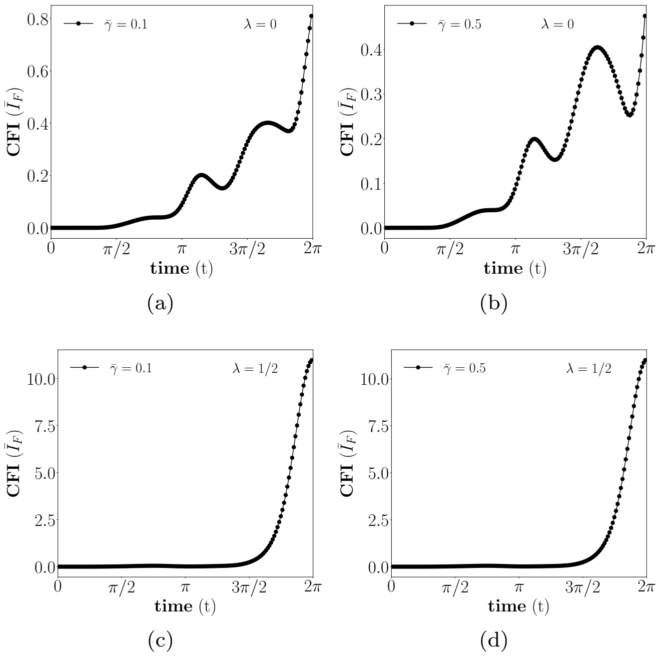

Supplementary Figure 2: Plots showing the classical Fisher information for measurements on the leaking photons. (a) and (b) show the CFI for position and momentum measurements respectively with $\gamma = 0.1$, and (c) and (d) show the CFI for position and momentum measurements with $\gamma = 0.5$. The additional parameters were set to $\bar{k} = \bar{g} = 1$, $\beta = 1$ and $\alpha = 1$.

In Supplementary Figure 2, we present additional results from numerical computations with $\gamma = 0.1$ and $\gamma = 0.5$, including both the position and momentum quadrature measurements for each run. As before we used parameters $\bar{k} = \bar{g} = 1$ and $\alpha = 1$. We note that a larger γ does not significantly impact the extracted CFI, which is to be expected as the information about g is generated by the motion of the oscillator and is transferred to the light field at a set rate not influenced by γ. With the choice of $\alpha \in \mathbb{R}$, we obtain largest I_{F} for momentum measurements, although at $t = 2\pi$ we do not see a complete reduction to zero for position measurements. Clearly the addition of the environment changes the behaviour of the CFI. The difference in I_{F} for the position measurements between the two runs is possibly due to numerical errors.

Supplementary Note 4

In the main text, we stated that the Fisher information for atom interferometry is equal to $n^2 T^4 k_{\mathrm{C}}^2$ and that optomechanical systems are enhanced by a factor of $\xi_{\mathrm{FP}} \sim c^2/(nL^2\omega_m^2)$ in comparison. Here we detail the derivation of the expression and the enhancement factor.

In atom interferometry, we prepare the atoms in a superposition of a ground state $|g\rangle$ and an excited state $|e\rangle$, such that the full state becomes $|\psi\rangle = (|g\rangle + |e\rangle)/\sqrt{2}$. Photons are then used to separate the two states by momentum transfer, causing them to take two different paths through a gravitational potential. We then assign a potential gravitational energy $mg\Delta x$ for the excited state, where Δx is the difference in height between the two paths. The phase accumulated by the excited state is then equal to $e^{img\Delta T/\hbar}$, where m is the atomic mass and T is the time of flight. We must now determine Δx. Ignoring any geometric factors associated with the paths, we assume that the distance roughly depends on the atoms' velocity v and their time of flight T. That is, we let $\Delta x \sim vT$. The total velocity is determined by the momentum transfer from the photons in the laser pulse, and is therefore proportional to the number of photons n. The momentum carried by one photon is given by $\hbar k_{\mathrm{C}}$, where k_{C} is the wavevector of the photon (which we take to be the same as the wavevector of the photons in the cavity). Thus, assuming that each photon transfers all of its momentum to the atom, we find that

$$\Delta x \sim vT \sim \frac{n\hbar k_{\mathrm{C}}}{m} T. \tag{4}$$

If we insert this into the expression for the phase and apply it to the state, we find

$$|\psi\rangle = \frac{1}{\sqrt{2}} \left(|g\rangle + e^{ingk_{\mathrm{C}}T^2} |e\rangle \right). \tag{5}$$

Calculating the quantum Fisher information for this state is straight-forward. We find that

$$\begin{aligned} H_{\mathrm{Q}} &= 4\left(\langle \partial_g \psi | \partial_g \psi \rangle - |\langle \partial_g \psi | \psi \rangle|^2\right) \\ &= 4\left(\frac{n^2 k_{\mathrm{C}}^2 T^4}{2} - \frac{n^2 k_{\mathrm{C}}^2 T^4}{4}\right) \\ &= n^2 k_{\mathrm{C}}^2 T^4. \end{aligned} \tag{6}$$

Since k_{C} has dimension m^{-1}, this expression has the correct units of $\mathrm{s}^4\mathrm{m}^{-2}$. In terms of scalability, we note that this expression surpasses the Heisenberg limit in terms of the number of photons n, and that it is highly dependent on the time of flight T.

We can now compare this with the optomechanical Fisher information for the Fabry-Perot cavity. The explicit Fisher information with k_{FP} inserted into Eq. (11)

given by

$$H_{Q,\mathrm{FP}} = \frac{32\pi^2 n \cos^2\theta}{\omega_0^6} \frac{\omega_\mathrm{C}^2}{L^2}. \tag{7}$$

where we have replaced $|\alpha|^2$ by n. To compare the two expressions, we let $\omega_m^4 \sim 1/T^4$, $\omega_\mathrm{C} = 2\pi c/\lambda$, and $k_\mathrm{C} = 2\pi/\lambda$. We set $\theta = 0$ for clarity and then divide them to find that

$$\xi_\mathrm{FP} = \frac{32\pi^2 n c^2 T^4/(\omega_m^2 \lambda^2 L^2)}{4\pi^2 n^2 T^4/\lambda^2} \sim \frac{c^2}{n\omega_m^2 L^2}.$$

This is the enhancement factor mentioned before. A similar analysis can be performed for the levitated nanospheres.

Supplementary Note 5

The QFI obtained in Eq. (11) scales linearly with the number of photons $|\alpha|^2$, and so does not allow us to reach the so-called Heisenberg limit. To achieve a quantum speedup in the sensitivity Δg, one must instead show that the Fisher information scales with the number of photons squared, that is $|\alpha|^4$. As we have shown, a coherent state will not achieve this, but a highly non-classical superposition of two Fock states will be more successful. We will show this by considering the following state

$$|\varphi\rangle = \frac{1}{\sqrt{2}} \left(|0\rangle + |n\rangle\right) |\beta\rangle\,, \tag{8}$$

where n denotes the number of photons. This state evolves under $\hat{H}_\mathrm{G}$ into

$$\begin{aligned} |\varphi(t)\rangle = \frac{1}{\sqrt{2}} \Big[& |0\rangle \left|\beta e^{-it} - \bar{g}\eta\right\rangle \\ & + e^{i(\bar{k}^2 n^2 - 2\bar{k}\bar{g}n)\tau}\, e^{\bar{k}n(\eta\beta - \eta^*\beta^*)/2} \\ & \times |n\rangle \left|\beta e^{-it} - (\bar{k}n - \bar{g})\eta\right\rangle \Big]. \end{aligned} \tag{9}$$

Finally the QFI obtained at $t = 2\pi$ for this state is given by

$$\begin{aligned} H_\mathrm{Q}(2\pi) &= 4\left(\langle\partial_g\varphi|\partial_g\varphi\rangle - |\langle\partial_g\varphi|\varphi\rangle|^2\right) \\ &= 4\left(\frac{\partial\bar{g}}{\partial g}\right)^2 \left(8\pi^2\bar{k}^2 n^2 - 4\pi^2\bar{k}^2 n^2\right) \\ &= 16\cos^2\theta \frac{m}{\hbar\omega_m^3}\pi^2\bar{k}^2 n^2, \end{aligned} \tag{10}$$

where the appearance of n^2 indicates that the standard Heisenberg limit has been surpassed.

While these states are very difficult to prepare, using $\bar{k}_\mathrm{FP} = 2.30$ for the Fabry-Perot mirror and cavity systems, and choosing a single photon with $n = 1$ gives us a sensitivity $\Delta g \sim 1.5 \times 10^{-11}$ ms^{-2}. Although it is not as high as the Δg obtained for coherent states, the increased power of the scaling offers interesting opportunities as technologies improve.
